# Remedies and Their Uses

**Published:** 1906-11-17

**Authors:** 


### Remedies and their Uses.

### Thiosinamine and Fibrolysin.

Thiosinamine is not a trade name; it is a shorter chemical name of the body known as Allyl Sulpho Carbamide. Carbamide, $CO<\begin{smallmatrix}NH_2\\NH_2\end{smallmatrix}$, is well-known as urea; a corresponding sulphur compound is known to chemists under the name of Sulpho Carbamide or Thio-urea $CS<\begin{smallmatrix}NH_2\\NH_2\end{smallmatrix}$. If Allyl, a radicle having the formula $C_3H_5$, be substituted for one of the hydrogen atoms $CS<\begin{smallmatrix}NHC_3H_5\\NH_2\end{smallmatrix}$ or Allyl Sulpho Carbamide is produced. In actual practice this substance is prepared by the action of allyl mustard oil $CS:N.C_3H_5$ on ammonia $NH_3$, the two uniting directly to form Thiosinamine. The body thus produced is a crystalline substance with a bitter taste, soluble in water and more so in alcohol and æther. It was first employed by Hebra, in 1892, in the treatment of lupus. A local reaction occurred on the injection of a small amount of the drug, without any general symptoms, and a notable diminution in the size of the scar tissue. Since then it has been employed in a large number of instances to reduce the amount of fibrous tissue in scars, contractures, adhesions, etc. The greater part of the drug appears unchanged in the urine, but a small portion is decomposed and is excreted by the lungs, probably as ethyl sulphide, a body possessing a characteristic alliaceous odour. It exercises in some cases a diuretic action, as might be expected from its close chemical affinity to urea. The urticaria and local anæsthesia sometimes produced are due to damage to local nerve endings, and are the result of the injection *per se* rather than the drug injected. Large doses sometimes cause somnolence. A case is recorded by Brinitzer (B.K.W. xliii. 4. 06.) in which scleroderma was being treated by Thiosinamine, but the patient developed prostration and high temperature. Fibrolysin produced a like effect, but small doses were subsequently given, and gradually toleration was established. This, however, appears to be a very rare occurrence.

The drug may be given by the mouth, but the effects are much less notable. Usually it is given subcutaneously at any convenient spot. A 15 per cent. to 20 per cent. alcoholic solution may be used, or a 10 per cent. watery solution with glycerine, which should be warmed before use. The latter causes less pain on injection, but is not quite so stable. One cc. (17 minims) of the solution twice a week may be given, and the dose may be rapidly increased to about 2cc. Munn has given it in the form of a 20 per cent. plastic needle. The cases in which the remedy has been said to give good results are fibrous stricture of the œsophagus and pylorus, stricture of the urethra, Dupuytren's contracture, fibrous synæchiæ in the eye, and in certain middle ear diseases. It is said in Dupuytren's contracture and in some urethral strictures the results were not permanent, but that the drug was a useful aid to other forms of treatment.

The way in which Thiosinamine acts has not been determined. It produces firstly a destruction and then an increase in the leucocytes. It has been thought to owe its irritating vasodilator effect to the mustard oil to which it is allied. It may thus act by improving the circulation and carrying new blood elements through the fibrous tissue. Other authors connect its action with some chemotactic power.

A disadvantage attending the use of Thiosinamine is the painful nature of the injections, due to their alcoholic vehicle. Merck, of Darmstadt, has therefore introduced a compound of sodium salicylate and Thiosinamine which is more soluble in water, and has precisely the same physiological effect. This substance, however, is very unstable when exposed to light and air, and is therefore sold in capsules, ready sterilised for hypodermic use. Each capsule contains ♏ 37 of the solution and corresponds to .2 gram (gr. 3) of Thiosinamine. (Two-thirds of the Fibrolysin molecule consists of Thiosinamine.) The injections may be made every day, every other day, or every third day, according to the nature of the case, and may be accompanied by other forms of treatment, such as massage, etc. The total number of injections necessary varies—fifty may have to be given. For rapid action the injections may be intravenous, but ordinarily Mendel (Therap. Monatsh. Ap. 1905) strongly recommends intramuscular injections into the gluteal region. This method is much easier to carry out, is absolutely painless, and the efficiency is proved by the appearance of the characteristic garlic-like odour in the breath about twenty minutes later. This shows that the fibrolysin is decomposed into Thiosinamine and a salicylate in the tissues. The subcutaneous method may be used where it can be applied at the site of the lesion; it is likewise painless. The action of these substances on white fibrous tissue seems undoubtedly established in certain cases. The permanency of the result is the only point which still requires to be determined. At present a recurrence has only been noted after the use of Thiosinamine. In some cases the treatment has appeared to produce a general improvement in health, and in cases where the cicatrix or band of new fibrous tissue could be watched this has been found visibly to diminish. On the other hand the evidence as regards internal conditions, such as pyloric stenosis, must be considered as less conclusive, partly owing to uncertainties of diagnosis and partly to the impossibility of excluding the influence of general concomitant treatment.

